# The potential role of cultural and religious healing practices in shaping community vulnerability to highly infectious diseases in western Kenya

**DOI:** 10.1371/journal.pgph.0003228

**Published:** 2025-03-25

**Authors:** Naomi Wambui Ng’ang’a, Reuben Onkoba Momanyi, Caleb Chemirmir, Hazael Biwott, George Ayodo, Monica Orero, Damaris Ochanda, Sarah Ngere, Winnie Ogola, Titus Murundu, Geoffrey Munene, Zachary Misiani, Michael Ayabei, Richard Dimba Kiaka

**Affiliations:** 1 Kenya Red Cross Society, Nairobi, Kenya; 2 Jaramogi Oginga Odinga University of Science and Technology (JOOUST), Bondo, Kenya; 3 Masinde Muliro University of Science and Technology (MMUST), Kakamega, Kenya; 4 Kenya Medical Research Institute (KEMRI), Nairobi, Kenya; Southern Cross University, AUSTRALIA

## Abstract

Cultural and religious practices and beliefs have historically played a significant role in the management of disease outbreaks globally. This study explored how such beliefs and healing practices shape the vulnerability of communities to highly infectious diseases in three border counties in western Kenya-Homa Bay, Bungoma, and West Pokot. Using an empirical qualitative research design, we conducted 45 key informant interviews with 13 traditional healers, 16 religious healers, and their 16 patients. We also held 6 focus group discussions with community members knowledgeable about cultural customs and practices, and 1 participatory inquiry workshop with health professionals and administrators. The findings indicated that traditional and religious beliefs and healing practices influence community vulnerability to highly infectious diseases in two main ways. Firstly, we identified a dualistic illness etiology that includes both biomedical and socio-cultural interpretations. Traditional and religious healers often served as the first point of care for unexplained illnesses or those unresponsive to conventional medicine, which could delay appropriate treatment and compromise safe handling in case of highly infectious diseases. Second, we found that traditional and spiritual healing practices pose certain risks. Practices such laying of hands, use of herbs and rituals involving slaughtering of animals enhanced contacts. The use of protective gear among healers was inconsistent and often absent due to cost or fears that it could undermine the patient’s faith in the healer’s powers. These practices can potentially predispose individuals to highly infectious diseases, enhancing transmission and symptom severity. To mitigate the vulnerability of border communities to highly infectious diseases, we recommend comprehensive strategies that address the intersection of vulnerability factors, including healing beliefs and practices. This may involve policy initiatives to integrate traditional medicine practices with the mainstream health system, thereby enhancing disease prevention and control efforts.

## Introduction

Cultural and religious beliefs, as well as practices, worldwide significantly influence the management of infectious disease outbreaks, where swift response and effective control are essential [1–3]. While some of these beliefs and practices can complement efforts to manage highly infectious diseases [4,5], others foster health-seeking behaviors that can undermine interventions. This can be experienced in situations where well-intended interventions by governments and emergency organizations conflict with communities’ cultural health practices. Recent examples, such as the responses to COVID-19 [6–9] and Ebola Virus Disease (EVD) [10,11], highlight these conflicts between community practices and governmental measures to manage infectious diseases.

The containment of these highly infectious diseases often requires stringent measures that disrupt daily life, including restrictions on social interactions, movement, and the suspension of important cultural ceremonies and rituals [6]. Through policy actions such as lockdowns and government-mandated safe burial procedures, governments and health agencies take control of areas traditionally governed by cultural and religious doctrines. Communities may resist these interventions, as they challenge deeply ingrained cultural and religious roles in everyday life. The resistance, whether active or passive, hampers the effectiveness of governmental interventions and compromises health outcomes.

Blaming and victimizing communities’ cultures and religions for conflicts with emergency health interventions is common [12,13]. Governments often view unique cultural and religious traditions as obstacles to health interventions during outbreaks of highly infectious diseases [14].

Such policy attitudes exacerbate tensions between formal interventions and cultural practices. Medical anthropologists stress the need to actively consider how local cultures interact with health concepts to reconcile formal interventions with cultural practices [15,16]. That is, before asking a community to assume particular health habits, adopt new health behaviors, it is crucial to understand existing health-seeking practices, how they function and what they mean to people who practice them [17]. This calls for a paradigm shift from top-down health interventions to a community-centered approach, where local health behaviors and their rationalizations are central to analysis. Importantly, research should explore how cultural practices shape and are shaped by formal health interventions, especially in the context of highly infectious diseases when socio-economic aspects of the communities are often under pressure and health resources are overstretched. In Africa, many communities use non-biomedical treatments, including herbal medicine and spiritual healing, to promote health [18]. Reasons for this range from inadequate access to healthcare facilities and the high costs of biomedical treatments to traditional and religious beliefs about disease and health. Despite the growing promotion of Western medicine, traditional and religious healing remains an important health resource. Many people seek traditional and religious healers as their first option when dealing with highly infectious diseases like Ebola [19]. Experience in Africa shows that when disease intervention programs fail to acknowledge and collaborate with indigenous beliefs and practices, they often remain ineffective and fall short of their goals [20,21]. The World Health Organization (WHO), in its *Traditional Medicine Strategy 2014 – 2023,* emphasizes the global importance of these alternative approaches to health [22].

This shift moves away from rejecting cultural and traditional healing practices, recognizing that these approaches are valued by many people and often used in combination with biomedical treatments. To understand why infectious disease programs sometimes fail and how to improve them, research and interventions must account for the culturally embedded health-seeking behaviors of the communities they aim to serve.

Drawing on fieldwork conducted in selected communities in western Kenya, this paper analyzes how traditional and religious beliefs intersect with concepts of disease, health, and healing. It addressed two research questions: 1) How do traditional and religious beliefs and associated practices influence, and are influenced by, highly infectious diseases and their management? 2) What are the implications of this influence for effective disease management and ensuring good health during outbreaks?

We focused on the social and behavioral aspects of infectious diseases within communities where non-Western cultures and religions strongly influence social life and understanding of health. The religions we analyzed in the study locations constituted mainly those churches that combine African traditions and elements of Christian beliefs and doctrines. We drew on theoretical insights from medical anthropology and cultural psychology to explore how culture, mind, and biology interrelate in the management of infectious diseases. Schaller and Murray reported that community experiences and responses to highly infectious diseases are often enmeshed in sociocultural beliefs and practices that mediate interactions with the physical and metaphysical world [23]. Similarly, Baye et al., highlighted the role of cultural factors in shaping social behavior and its impact on spread of highly infectious diseases [24].

We utilized the concept of “the looping effect” to argue that cultural and religious beliefs create dynamic interactions with biological diseases, influencing perceptions of treatment and health outcomes [25–28]. The looping effect refers to the process by which cultural practices and beliefs form feedback loops with biological phenomena, such as disease, reshaping both the meaning of treatment and the overall goal of good health [26]. Looping effect theory posits that while people’s beliefs do not directly intervene on the virus itself, they can alter critical features of highly infectious diseases, such as infection rate, severity, and resistance to drugs through virus mutations [24]. In the context of highly infectious diseases, understanding these loops can provide critical insights into the barriers and enablers of cultural knowledge and behaviors in preparedness and emergency response planning. This framework allows us to connect religious and traditional healing preferences in the communities studied and understand how health-seeking behaviors form looping effects with both the diseases and the responses to them. Instead of viewing culture (metaphysics) and disease-causing pathogens (physical entities) as parallel existences, we analyze their indirect mutual interactions [24].

We proceeded to describe the methodology including providing a description of the study sites and data collection and analysis methods. We then presented our findings and discussed them in light of their implications to literature, policies and the practices for intervening on highly infectious diseases outbreaks. We finally drew some conclusions and suggested some recommendations.

## Materials and methods

### Study settings

Fieldwork was conducted between September and November 2023 in western Kenya in the counties of Homa Bay, Bungoma, and West Pokot. In the context of highly infectious diseases, the three counties share a porous border with neighboring Uganda and Tanzania which have in recent times seen outbreaks of Ebola Virus Disease [29,30] and Malburg [31] respectively. Homa Bay County has also struggled with multiple outbreaks of cholera in recent years. Moreover, these counties are characterized by strong traditional belief systems that influence health seeking behaviour [32–35]. The counties selected practiced Islam, Christianity, African traditional religion and African initiated churches such as Legio Maria and Roho Israel. There is also a persistent practice of unique religious beliefs including deifying humans and asserting that healing can possibly be achieved through faith and religious rituals, e.g., in Bungoma. These religious beliefs influence health seeking for communities in ways that impinge on management of highly infectious diseases, including Ebola Virus Disease (EVD). Lastly and equally important, these counties were selected for surveillance and health education by a national taskforce on EVD following the 2022 outbreak in Uganda.

The counties of Homa Bay, Bungoma, and West Pokot in Kenya each have distinct ethnic identities.

Homa Bay, located on the southern shore of the Winam Gulf of Lake Victoria in western Kenya, is predominantly inhabited by the Luo ethnic group. As of 2019, the county had a total population of 1,131,950 and is divided into 8 sub-counties and 40 wards [36]. The county is home to a variety of religious practices, including African traditional religions.

Bungoma County, situated in western Kenya, is predominantly occupied by the Luhya community. The Bukusu people, a sub-group of the Luhya community are the majority, other sub-groups include, the Tachoni, Batura, Sabaot, Iteso and Bongomek. Like Homa Bay, Bungoma also has a diverse range of religious practices, including African traditional religions. From the 2019 Kenya Population and Housing Census report, the county had a total population of 1,670,570, divided into 9 sub-counties and 46 wards [36].

West Pokot County, located further west, is predominantly inhabited by the Pokot people, a sub-tribe of the Kalenjin ethnic group. As of 2019, the county had a total population of 621,241, divided into 4 sub-counties and 20 wards [36]. The county, too, exhibits a range of religious practices, including African traditional religions.

### Study design

The study employed a qualitative study design using anthropological data collection approaches. The methods for this research study involved a combined approach of Key informant interviews (KIIs), Focus group discussion (FGDs) and participatory inquiry workshop. The KII guide were broad, less structured, open-ended set of question organized in thematic areas. FGD guide were semi-structured providing respondent with the flexibility to express their opinions. Participatory inquiry workshop guides were broad and open ended to allow discussions among group members The stakeholders for KII and FGDs were religious healers, traditional healers, patients of religious and traditional healers and community members while stakeholders for participatory inquiry workshops included government public health officers, Non-Governmental Organizations (NGOs) and Community Based Organizations (CBOs), Community Health Promoters (CHPs) and local chiefs from the national administration office.

### Study participants, selection and data collection procedures

Snowballing sampling technique was used to select KII participants including, traditional healers, religious healers and their respective patients. Sample size was determined by the point of data saturation [37]. Using this approach, saturation was assumed when no new information emerged in the process of data collection and data began to replicate [38]. In accordance to previous qualitative studies, data saturation occurs within 12 interviews or even lower depending on the scope and objectives of the study [39,40]. While our initial participant estimates were based on typical saturation points in similar studies [41,42], full saturation was reached on a sample of 45 participants; 13 traditional healers, 16 religious healers and 16 patients of traditional and religious healers. This sample size was considered sufficient, as research indicates that between 16 and 24 interviews are typically required to achieve meaning saturation. Meaning saturation indicates that the researcher has “understood everything” [40].

Participants were identified through chain referrals with individual interviews conducted in health facilities or the respondents’ homes depending on the comfort of the respondent. Interviews were conducted in a language that the respondent was most comfortable with. Whereas in West Pokot County the interviews were translated, in Homa Bay and Bungoma, the researchers were able to directly conduct interviews in Dholuo and Swahili respectively.

FGD guides were semi-structured providing respondents with the flexibility to express their opinions. A total of 6 FGDs each (8-12 participants) were conducted with community members who are very knowledgeable about the local traditions and customs in the three counties. Each FGD was of a homogenous gender in that women FGD was separated from Men FGD due to cultural norms which could limit participation of female gender in a mixed discussion. The respondents were brought to a central point where sessions were conducted. The FGDs investigated a set of issues on sociocultural and religious healthcare seeking on highly infectious diseases. In Homa Bay, Dholuo was used to facilitate the discussions, in Bungoma Swahili was used to facilitate the discussions and lastly in West Pokot a translator assisted the moderator to translate questions into Pokot.

Participatory inquiry workshops were used to collect data and information from public health promotion stakeholders in each county. These stakeholders included public health officers from government, Non-Governmental Organizations (NGOs) and Community Based Organizations (CBOs), Community Health Promoters (CHPs) and local chiefs from the national administration office. The workshop was conducted in central locations. Participants were divided into groups depending on their category. Each group had a moderator and a note taker. They were given a list of questions to discuss and generate detailed group responses. After about an hour of group discussions, participants gathered in a plenary to discuss their points. Points were interrogated by participants and adjustments made as was relevant and agreeable to participants.

All data collection tools including focus group discussion and key informant interview guides, as well as those for participatory inquiry workshops were collaboratively developed by all authors. Two joint meetings were conducted to review, harmonize and validate data collection tools before they were submitted to an ethics institutional review board. Trial was conducted five days to data collection where changes were proposed to the various tools. These changes were further incorporated in the tools and resubmitted to the ethic review board for further consideration and approval. Table 1 below outlines the category and number of participants in the study.

**Table 1:** Description of research participants.

Data collection method	Categories of participants	Location	Total	Male	Female
Interviews	Traditional healers	All	13	7	6
		Homa Bay	4	2	2
		Bungoma	4	2	2
		West Pokot	5	3	2
	Religious healers	All	16	9	7
		Homa Bay	6	3	3
		Bungoma	5	3	2
		West Pokot	5	3	2
	Patients of traditional and religious	All	16	7	9
		Homa Bay	6	3	3
		Bungoma	6	2	4
		West Pokot	6	2	2
FGD	Community members	All	56	28	28
		Homa Bay	24	12	12
		Bungoma	16	8	8
		West Pokot	16	8	8
Participatory workshop	National administration Office(chiefs)	All	57	32	25
		Homa Bay	5	3	2
		Bungoma	5	5	0
		West Pokot	6	6	0
	Nurse/Clinical Officer	Homa Bay	4	2	2
		Bungoma	4	1	3
		West Pokot	4	1	3
	Community Health Promoters	Homa Bay	5	0	5
		Bungoma	3	0	3
		West Pokot	6	4	2
	Public health Officers	Homa Bay	7	3	4
		Bungoma	4	4	0
		West Pokot	4	3	1

### Data management and analysis

All discussions from KIIs, FGDs and participatory inquiry workshops were audio recorded and transcribed verbatim into English. The framework method was employed to analyze the data, using both inductive and deductive coding [43,44]. The authors jointly developed a coding framework organizing it into a spreadsheet matrix with numerous themes and sub-themes under which relevant excerpts from the transcripts were entered. Irrelevant codes were deleted while adding emergent codes to the matrix as needed. The transcripts were repeatedly reviewed to ensure thorough and consistent coding, following similar practices applied in other ethnographic studies conducted in Mombasa, Kisumu, Nairobi, and Kwale counties in Kenya [45,46]. Emerging themes were collated, collapsed, defined and interpreted as results.

### Ethics Statement

Ethical approval No. MMUST/IERC/185/2023 was granted by the Masinde Muliro University Institutional Scientific Ethics Review Committee (MMUST-ISERC). Respondents’ informed consent were sought in writing before interviews. Under the informed consent, the respondents were provided with all the pertinent information about the study to guide them in making a decision on whether to participate in the study or not. The information provided in the consent forms included: the purpose of the study, the procedures to be followed; and the benefits/risks of this study. There was no foreseen harm to the participants however, participation in the study was completely voluntary, and respondents’ confidentiality has been maintained such that no information in the processed data and publications can lead to the identity of an informant. Data collection took 3 days in each of the 3 counties, where in Homa Bay the team collected data from 25^th^ to 27^th^ September, 2023, and in Bungoma and west Pokot data collection was conducted between 11^th^- 13^th^ October 2023.

## Results

Between September and October 2023, interviews were conducted with 13 traditional healers, 16 religious healers, and 16 patients who had consulted traditional or religious healers in Homa Bay, Bungoma, and West Pokot counties. Among the key informants, 51.1% were male and 48.8% were female.

Additionally, a participatory workshop was held to collect data from various public health promotion stakeholders. Participants included chiefs from the national administration office (28.1%), public health officers (26.3%), community health promoters (24.6%), and nurses and clinical officers (21.1%). These workshops provided valuable insights into local health practices and challenges faced by different communities. See Table 2 for demographic summary of the participants.

**Table 2:** Participants characteristics by gender.

Data collection method	Categories of participants	Total	Male	Female
		**N**	**n (%)**	**n (%)**
KII	All	45	23 (51.1%)	22 (48.8%)
	Religious healers	13	7 (53.8%)	6 (46.2%)
	Traditional healers	16	9 (56.3%)	7 (43.7%)
	Patients of religious and traditional healers	16	7(43.7%)	9 (56.3%)
FGD	Community members	56	28 (50%)	28 (50%)
Participatory workshop	All	57	32 (56.1%)	25 (43.9%)
	National administration Office (chiefs)	16	14 (87.5%)	2 (12.5%)
	Nurse/Clinical Officer	12	4 (33.3%)	8 (66.7%)
	Community Health Promoters	14	4 (28.6%0	10 (71.4%)
	Public health Officers	15	10 (66.7%)	5 (33.3%)

### Traditional and religious illness and healing beliefs

#### Beliefs on causes of illness.

There exists a dualistic illness etiology in Homa Bay County, Bungoma and West Pokot County. Both traditional and religious (especially those from African Instituted Churches) spheres in the county consider diseases to be associated with biomedical and sociocultural causes. Using a biomedical lens to explain disease etiology, religious and traditional healers, their patients and participants of FGDs associated some diseases to viruses, bacteria and dirt and poor hygiene. For example, cholera was associated with poor sanitation, while the COVID-19 was mentioned to be caused by coronavirus.

Diseases such as diarrhea and vomiting are believed to be infectious which are caused by biomedical agents. Infectious diseases are believed to spread fast through contaminated food and water.

Socio-cultural illness etiology is largely associated with customs and beliefs. Witchcraft was mentioned as one of the causes of illness. Witchcraft distinguishing signs are diverse but largely associated with abnormal illness such as mental disorder, bloody vomit, severe headaches and severe stomach aches. Among discussants in a Focus Group one of the respondents says that:

*…then for example when a child is looked at badly (evil eye) with a witch(iro), then he is taken to the hospital and injected, he will not be well. So, some people believe if I run to the religious healer, they will be well.*

FGD2, Homa Bay.

A respondent explains elaborately how a child can get sick from an ‘evil eye’ in the excerpt below. It is believed that diseases caused by an ‘evil eye’ or witchcraft cannot be treated in the hospital also referred to as *non-hospital diseases*. *Non-hospital* cases are treated by either traditional medicine or by religious healers. The rationale is that if the disease is treated in the hospital the sick person will not recover from the sickness.

*For example, there is a particular illness that forces you to go for that type of treatment. When someone looks at a child who is breastfeeding with bad eyes; so there are people with bad eyes that will make this milk turn into something else in this child’s stomach. So a hospital won’t help you in this case. So there are people who can scratch the bad milk from inside the child (saro) hence this traditional healers, there are places where they come in handy like such not that they don’t help completely.*

FGD2, Homa Bay.

Disease etiology is also arrived at when a particular disease does not respond as quickly as desired to biomedical treatment. Sometimes when traditional or religious healing is sought, the rationale is driven by non-response to biomedical treatment. Those diseases that take too long to recover are believed to be caused by the power of *satan*. The *satanic* forces are believed to be inflicted by humans in a way of punishing the inflicted person/or when they are envious of the person. In Homa Bay a respondent stated that:

*A sickness that has been treated and has taken even 3 months and it is still that one sickness, it goes and comes back and goes and comes back that now shows that satan is in there so we have to pray to God.*

Religious Healer KII1, Homa Bay.

A patient of a religious healer from Bungoma believes that she was not responding to biomedical treatment because her illness was inflicted by an ‘evil eye’. The respondent believed that an envious person cast a spell of bad luck on them and made them severely ill with a disease that did not respond to conventional medicine.

*What I can say is “hasad” someone is not happy with your progress and looks at you with an evil eye because they are not happy with your growth. “Hasad” is bad luck.*

Patient of a religious healer KII2, Bungoma.

In West Pokot a respondent attributed illness causation to supernatural forces that is caused by supernatural forces emanating from the river. When a respondent was asked what causes pneumonia their response was as illustrated below.

*This disease originates if you cross rivers with supernatural thunders as we were told long time ago. And sure this supernatural being are there because if you cross the river and it doesn’t like you it will throw some soil at you. Last year it attacked some of our children in River chematong where they droned and they were searched for a very long time until one of the person identified them and for sure this supernatural thunders may exist.*

Traditional Healer KII3, West Pokot

Some respondents believed that the departure from indigenous traditions such as their traditional food practices causes the community illnesses. They believe that the food they consume currently makes them susceptible to illnesses.

*In the past people never used to get sick from these illnesses they used to take this vegetable called mhalaha, it helped their body to be strong, the soup from the cattle heads, they never used to be sick, they used to eat traditional food and now we eat meat from treated cattle, they put chemicals in milk we drink and the diseases are caused by these things.*

FGD1, Bungoma.

Traditional medicines are believed to protect someone from illnesses failure of using these drugs are causing illnesses in people.

*If you are used to taking traditional medicine, it is very hard for you to contract the diseases because the medicine washes all the blood veins you cannot get infectious disease. It helps you.*

*FGD1, Bungoma.*

#### Traditional and religious healing beliefs.

Traditional, religious and spiritual beliefs were noted to offer support and meaning to those coping with health challenges. Most of our respondents who went to traditional or/and religious healers were comforted and reassured of healing especially in instances where conventional treatment failed. In religious healing, faith was paramount for healing to take place. The decision to choose traditional or religious healing was dependent on individual choice.

*The traditional healers are there hence they treat illnesses that can’t be treated in the hospital, very many illnesses defeat doctors in the hospital hence the traditional doctors are the ones that clear them and once you take your patient there, they begin to drink porridge and they start speaking.*

Patient of traditional healer KII4, Bungoma.

In Christianity the doctrines in these churches emphasize the possibility of God giving healing powers to members of the congregation. They receive the healing powers through the holy spirit. Although the doctrines may differ in some areas, these churches share foundational beliefs in some diseases occurring because of curses, sins of witchcraft. Diseases associated with these cultural processes were reported by religious healers to be often associated with strange signs and symptoms that are diverse. One respondent states that God heals because everything happens in his power and therefore healing also comes from him.

*Everything happens through the power of the holy spirit or God. We believe in prayers and the Religious Healer can pray and we believe through the strength of Jesus Christ the patient is healed.*

Patient of a religious healer KII2, Homa Bay.

The religious healer therefore becomes the vehicle in which healing is performed but God is the healer as stated by a Bungoma respondent

*We do not say that somebody heals, it is God who heals not an individual. Maybe there is that bit of a praying, there is that bit of reading the verses of the Quran to get that healing from God.*

Patient of a religious healer KII2, Bungoma.

The Christian religious healers, largely consist of African Instituted Churches, e.g., *Roho* and *Legio Maria* churches. These churches combine Christian teachings with Luo customs to advance spirituality. Therefore, it is no wonder that some traditional healers mentioned that some religious healers refer patients to them to collect medicines for the further treatment and cultural advice or send to the hospital for further treatment. Religious healers who were interviewed believe that their powers come from God. It is from these powers that they are able to discern *hospital* and *non-hospital* illness and refer accordingly.

*God gave me some gift that by just seeing you the way we have sat and when I just start praying for you, I get a vision of what’s going on with you and even in your family. So that’s why I have to do the checking (diagnosis)for me to know the disease was caused in this way and what brought it. And if it happens that the disease was caused by a human, then we will pray to God. But if it’s just a disease which have just come up, then I will send you to the hospital.*

Religious Healer KII5, Homa Bay.

Religious healers are cognizant of the extent of their ability to treat diseases. Using their judgment and powers given by God traditional healers use a combination of spiritual healing and traditional medicine. In other cases, they refer their patients to the hospital especially for infectious diseases because they appreciate the severity of the disease.

*The patient was weak and had persistent cough accompanied by pain in the lungs. When he came, I prayed for him and after one week, I sent her to the hospital to check, the tests showed he had no TB.*

Religious Healer KII3, West Pokot

### Traditional and religious healing practices

#### Religious healing practices.

Religious healing involves rituals that are accompanied by prayer, laying of hands and consecration of the body using *holy water*. According to religious healers and their patients who were interviewed, *holy water* can be sourced through different means. Communities living near large water bodies such as the ones from Homa bay, water bodies and their water are significant in healing practices. the practice include fetching water from Lake Victoria boiling and consecrating it through prayer by a religious leader with healing powers. *Holy water* can also be drawn from water bodies with religious significance or mythical in Luo cultural cosmologies. A religious healer from Homa Bay explains how she prepares *holy water* in the excerpt below:

*we boil the water as we pray asking God to bless the water to turn into medicine. So, when I sprinkle in his body you see the body shakes. It sends away the devil. So ill pray for you that way and after that you’ll be healed and taken by your family members.*

Religious healer KII6, Homa Bay.

In Islam the religious practice involved citation of specific Quran verses, burning incense and praying over the sick person. This ritual is repeated until the sick person recovers.

*The prayers happened in our home but the first time he burnt incense in his home and my father did the same, he is also a Maalim. It’s just incense, while reading the Quran.*

Patient of a religious healer KII2, Bungoma.

In West Pokot religious healing involves prayers only, the religious healer lay their hands on the sick person and if they have faith in God then they are healed.

Some processes of administering healing that were mentioned involved body washing, where the healer washes the patient’s body using *holy water* or is washed using a cocktail of herbs mixed with water. The processes involve direct body contact between the healer and the patient.

*when they come, I will boil that water as I pray vehemently and after that, I first wash them with it after adding salt into it and a bit of sugar to stop the bleeding and can be done three or four times then drinks. He begins to urinate the infections like they urinate blood. At that time, I’ll be praying as I am washing him until the minute he arises.*

Religious Healer KII6, Homa Bay.

#### Traditional healing practices.

Traditional healers in Homa Bay, Bungoma and West Pokot County are similar and diverse in nature. In the 3 counties they differ depending on their specialty on the kind of diseases they treat. There are those dealing with broken bones and displaced joints, some deal only with mental disorders, others deal with snake bites. There are those who deal with diseases associated with witchcraft, while others deal with diseases that are clinical in nature such as measles, asthma, stomach aches etc. Healing for diseases associated with witchcraft and those that are clinical in nature are the ones that have direct implications on management of highly infectious diseases. A patient of a traditional healer explains how he was treated when he could not find treatment for an illness that was associated to socio-cultural etiology.

The treatment included traditionally cutting through the flesh *(chanja)* and inserting herbal medicine.

*Because he saw my body swelling, my leg swelling, my chest swelling, he just started making small cuts and inserting medicines and those medicine that got into my body, made me feel pain when they were entering hence from there, it just took me one week and I started seeing my body going back to normal, he started from there then he gave me another one for drinking and I got well after also the herbs which he cut into my body.*

Patient of traditional healer KII4, Bungoma.

Traditional healers also treat illnesses that can be bio medically defined such as malaria, Typhoid, TB, hepatitis B, sexually transmitted infections and many others. They report that they are able to know what disease the patient has by report from patient, observation of symptoms or palpating the patient. The herbs used are used to specifically treat certain ailments. The treatments are sometimes accompanied by rituals such as slaughtering an animal.

Special ritual is made for protection against diseases in the recent past the communities of West Pokot were cleansed for protection against COVID-19. The ritual involves slaughtering a cow and burning it. Herbal medication is prepared and drunk in order to protect them from the illness.

*…during cleansing we curse these diseases so they can’t continue they stop there. So, the cleansing is like slaughtering a cow and burn it. Then there are drugs we take, there are others we drink and pour so the diseases don’t come to us in our village.*

FGD2, West Pokot.

#### The practice of administering healing.

Some processes of administering healing that were mentioned to be having the risks of predisposing healers and patients to infection included body washing, where the patient washes the body or is washed using a cocktail of herbs mixed with water. The processes involve direct body contact between the healer and the patient, inhalation, where the patient inhales medicines under a blanket. The blanket is often used by other patients as well; piercing or cutting skin or veins to suck out what was termed as “*bad blood”*, with the high risks of creating direct contact with patients’ body fluids; laying of hands by religious healers during prayers for the patient, which leads to direct body contact; sprinkling of holy water and medicine on the body of the patient. In some cases, the healers drink the medicine and hold it in their mouths from which they sprinkle to the face of the patient; and massaging the patient either while praying or applying medicine. In relation to highly infectious diseases, these processes share one thing in common – the contact with the body or body fluids which has the potential of enhancing the spread of viruses and other disease-causing pathogens.

*What happens is after dying, we as 3 female healers wash the body clean so that we avoid spread of diseases to those who come and had no idea what they were suffering from. When the others come, we advise them to avoid touching the person because they were sick.*

Religious healer KII3, Homa Bay.

#### The practice of handling waste.

Interview data show that religious and traditional healers do not have the necessary knowledge and capacity to handle patients’ wastes. These wastes include vomit, blood, sputum, faecal matter, and general dirt from clothes. When asked how they dumped the wastes, most traditional healers mentioned that they dump them into a pit latrine in their compounds or bury them into the soil. Others dump or wash the wastes in water bodies that are communally used in the communities, e.g., the lake, rivers, or water ponds. For example, when the traditional healer, was asked how he cleans the blankets that he uses to provide inhalation treatment to his patients, he said:

*My wife must wash them in the lake. I also keep them in my house because it’s never anything threatening that can cause serious issues in the event the blankets are stored in the house or anything that has the potential of affecting my children.*

Traditional healer KII6, Homa Bay.

This example demonstrates lack of knowledge and capacity of handling waste from patients, as pathogens causing highly infectious diseases can leak through the poor waste-handling practices into the community leading to widespread infection and overstretching management measures.

#### Healing paraphernalia.

Traditional and religious healing have many paraphernalia depending on the kind of treatment given. These can range from containers for bathing, containers for drinking medicines, sharp objects for cutting or piercing the skin; blankets for inhalation; etc. Participants reported that there was widespread sharing of healing paraphernalia, e.g., the same basin or bucket for bathing patients or the one calabash for drinking medicines by different patients and the same blanket for inhalation processes. Healers justified the sharing of paraphernalia in many ways namely, that it is cumbersome for patients to be bringing their own materials as they come for healing services; and that some of the paraphernalia are consecrated or spiritually dedicated for use and this is also central to the healing of a patient. Sharing of healing paraphernalia has the risks of passing infections from one patient to another.

However, other healers asked the patients to bring their own personal effects like basin, cups and plates for use during their stay as reported in the excerpt below:

*aside, yes, he/she vomits there when it is done you tell their people now wash, they will bring their basin, if they come I tell them to buy their basin and utensils they will use to eat. He stayed three weeks and he was healed.*

Traditional healer KII1, West Pokot.

The preparation, storage and administration of the medicines demonstrate poor observation of hygiene. For example, traditional healer, described one of how he does his healing processes in the following manner:

*Yes, once you’re seated here and these used to belong to my grandfather. I then take the hoe and detach it from the handle together with a new broom […..]. Once I have it, I then instruct my client to open his/her mouth, then the medicine moves from the broom to the hoe and lands in the mouth.*

Traditional healer KII6, Homa Bay

#### Patient handling.

Traditional and religious healers are generally not trained in patient handling. They rely on their experience with various illness and patients. While their experience might be sufficient for the healthcare they give, their safety and that of their patients are not guaranteed. Most religious and traditional leaders as well as their patients mentioned that the healers usually do not use personal protective equipment (PPE) such as gloves, masks, and aprons. They handle their patients with bare hands and without protection against coughs, sneezing and spillage of body fluids such as blood. This predisposes them to highly infectious diseases in case their patients would be suffering from one. Some reasons were mentioned to be contributing to the lack of personal equipment were. First, some healers mentioned that they could not afford gloves, masks, aprons, and sterilizing agents, since they usually charge little money for their services or receive payment in kind. Another reason was the lack of knowledge that a disease could be highly infectious. Education and strategic awareness creation could help address this problem. A third reason was the need to demonstrate faith and power over ailments. This was especially true for some religious healers.

For the religious healers, handling patients without necessary protection is meant to sustain the faith of the patient, which is an integral part of the healing process. Wearing and using protective clothing or equipment reduces the faith of the patient hence can compromise healing. However, this demonstration of spiritual faith leads to direct body contact that can be counterproductive in case the patients or the healers had EVD or other highly infectious diseases like COVID-19. It creates physical and social chains through which the diseases can spread through communities to reach pandemic levels.

The risks posed by failure to use PPE during traditional and religious healing was known to some participants. For example, some patients were aware that when a traditional or a religious healer handles them without PPE, then they can be predisposed to infections, especially of highly infectious diseases. To help illustrate this point, we consider response by a former patient of a traditional healer in the interview excerpt below:

*Yes, it would spread fast because she was handling me without gloves, gumboots, masks, she just put on her normal clothes, she had no apron the way nurses usually have one when delivering a baby, all these would have facilitated the spread of this disease.*

Patient of a traditional healer KII3, Homa Bay.

Some traditional healers reported using PPEs in their healing activities. For example, a traditional birth attendant mentioned that she wears gloves when massaging pregnant women and when they accidentally come on labour and give birth at their homes she ensured that the birthing conditions was clean The interview excerpt below helps to illustrate this point:

*You must have clean water and soap for washing hands before and after. The patient also has to be clean. The days we used to assist mothers to give birth, when cutting the umbilical cord, someone can give birth to a baby who is not very clean, so you have to wash your hands properly.*

Traditional Healer KII1, Homa Bay.

These relatively safe practices were reported to be as result of the collaboration between public health facilities and traditional birth attendants in the county. That the traditional birth attendant in the excerpt above intentionally ensures cleanliness and personal protection for herself and the patient is a good sign that increased awareness and education about safety during patient care can be fruitful endeavour.

## Discussion, conclusion and recommendations

The intersection of social, cultural, and religious forces with health has historically shaped patterns of disease exposure, health-seeking behaviors, and the adoption of interventions [47]. In the context of highly infectious diseases, such culturally mediated behaviors are often influential in shaping transmission dynamics within socially organized populations [24]. Cultural beliefs and practices, along with the health-seeking behaviors they inspire, create a looping effect that can impact disease outbreak prevention and response strategies [25,26]. This paper was set to investigate the role of traditional and religious beliefs and practices in mediating responses to highly infectious diseases in western Kenya. Further, we aimed to show how the analyses elucidate emergence of looping effects from the interaction between culture and disease, within the communities studied. In particular, the paper was set to show 1). How traditional and religious beliefs and practices influenced, and are influenced by, the management of highly infectious diseases; and (2) what implications of those influences had on the effectiveness of disease management.

The data showed two pathways through which cultural and religious practices of the communities in the studied area could intersect with the management of highly infectious diseases. That is through disease etiology and treatment practices. For disease etiology, the data showed the existence of a dualistic “cause” of diseases within the communities studied, where the causes of illness are attributed to both biomedical and sociocultural factors. This duality shaped two categories of illness: “hospital diseases” caused by biomedical factors and “non-hospital diseases” believed to arise from sociocultural influences. Diseases exhibiting abnormal symptoms, such as hemorrhagic fevers commonly associated with highly infectious diseases, are often linked to witchcraft, punishment for sins, or deviations from societal expectations. Earlier research in western Kenya on various diseases, e.g., HIV/AIDS [48,49], measles [20,50], and schistosomiasis [51,52] found that many people, especially amongst the Luo community, relied on cultural repertoires to explain the emergence and persistence of those diseases. Abubakar and others also found cultural perceptions on causes of diseases to be significantly influencing health-seeking behaviors of communities the Kenya’s coast [53].

Beliefs in the sociocultural causes of diseases was also rationalized and enhanced by past dissatisfaction with conventional medicine. An example involved women who believed that clinical treatments for measles prevented the disease’s characteristic rash from appearing, causing children to die from a treatable illness. This finding highlighted different ways of knowing the manifestations of measles between conventional doctors on the one hand and traditional healers/patients on the other hand. Similar studies, e.g., Haque et al., found that cultural and religious values were associated with lower trust in conventional medicine, which increased fear, conspiratorial thinking, and reduced willingness to adopt protective measures [42].

The dualistic perception of disease has adverse implications on diseases management. First, individuals who believe some diseases emerge through socio-cultural causes will first seek the intervention of traditional and religious healers for those illnesses. As Awah et al. observed, the reliance on traditional healing practices often delays appropriate management measures, such as diagnosis, isolation, and treatment, exacerbating the spread of disease and increasing the severity of symptoms in patients—particularly for highly infectious diseases [54]. As a result, there is often a critical delay in the deployment of effective public health interventions, which can accelerate the transmission of infectious diseases within communities.

Cultural beliefs about disease causation create a looping effect, where these beliefs influence health-seeking behaviors, thereby impacting disease outcomes. Diseases classified as “non-hospital” are perceived as treatable only by traditional or religious healers. Interviews with community members suggest that some highly infectious diseases are misclassified as non-hospital diseases, particularly when they presented unfamiliar symptoms. This misclassification positioned traditional and religious healers as the primary caregivers during disease outbreaks, further delaying timely access to formal healthcare systems. De Vries et al. observed similar patterns during the Ebola outbreak in Uganda, where traditional healing sites became primary epidemic spaces due to their role as first responders in treating patients [19]. The prioritization of traditional and religious healing during disease outbreaks thus compromised both treatment efficacy and the prevention of disease transmission. These gaps underscored the need for culturally-centered health education programs to improve the uptake of available interventions, particularly for highly infectious diseases. Health education initiatives could play an important role in shifting these cultural perceptions regarding the causes of these diseases [55]. However, as Chernet and Riako argued from their study on HIV/AIDS, health education initiatives could improve health-seeking behavior if they are culturally sensitive [56].

The analyses also showed that culture and diseases management intersected in the way in which traditional and religious healings are conducted. The data revealed that many healing practices were conducted in a manner that had the potential to enhance the spread of highly infectious diseases. For example, many interviews pointed out that healers did not have access to personal protective devices or failed to use them because their use could lower the confidence of patients in the treatment. These practices have a greater potential of exposing healers, their families, and patients to an increased risk of infection due to inadequate protective measures and limited knowledge of infection control. Examples have been documented during the Ebola outbreaks in West Africa [57–59] and Uganda [60], as well as COVID-19 in Western Kenya [6].

Despite these challenges, the popularity of traditional healing practices persists, often passed down through generations [42]. As traditional healers are frequently the first point of contact during disease outbreaks, it is essential to incorporate them into the broader public health response. De Vries et al. argued that public health interventions for diseases such as Ebola should neither stigmatize nor undermine these informal healers, as they play a central role in initial disease management strategies [19].

In conclusion, this study highlighted the need to recognize the looping effects between cultural beliefs and the management of highly infectious diseases. A transdisciplinary approach is essential to address the complex interaction between sociocultural, economic, and biomedical factors that drive disease outbreaks [61]. To achieve a more effective response to highly infectious diseases, public health communication and education must adopt a holistic approach that accounts for sociocultural factors [2,62,63]. Hussen et al. further emphasized the importance of cultural competency in designing health communication and education programs [64]. Acknowledging the central role of traditional healers in communities and engaging them in public health efforts is crucial for building trust and facilitating the timely adoption of preventive measures. However, few limitations of this study should be noted. First, while data saturation was achieved, meaning no new insights emerged in the study areas, the relatively small sample size may restrict the broader applicability of our findings especially since ethnic and religious practices are context-specific. Second, this study used the cultural intersections with health and healing to speculate what could happen in the event of an outbreak of a highly infectious disease like Ebola. More research is needed to further the understanding on the arguments presented.

Based on these findings, we make the following recommendations:

1. There is an urgent need for culturally centered public health education and awareness programs focusing on highly infectious diseases such as hemorrhagic fevers and acute respiratory diseases in western Kenya and similar contexts.
2. Public health communication strategies should be tailored to reflect existing disease etiologies within communities, integrating both biomedical and sociocultural perspectives.
3. Public health agencies and professionals involved in outbreak response should acquire cultural competencies specific to the communities they serve, allowing for more effective engagement and intervention.
4. Policymakers should explore the integration of traditional and religious healers into the formal healthcare system to ensure a coordinated and culturally sensitive response to disease outbreaks.

## Supporting information

S1 File This supplementary file contains research tools used to facilitate discussions with different stakeholders on relevant study themes. Legend: *Guide-for-FGD-Community-Members.docx-* Focused Group Discussion (FGD) guide for community members. *Guide-for-Religious-Healers.docx; Guide-for-Traditional-Healers.docx-* Key Informant Interview (KII) guides for traditional and religious healers. *Guide-for-Patient-of-Religious-Healer.docx; Guide-for-Patient-of-Traditional-Healer.docx-* Key Informant Interview (KII) guide for patients of traditional and religious healers. *Guide-Participatory-Enquiry-Workshop.docx-* Participatory workshop guide for stakeholders. *Informed-Consent-KRCS.docx-* Informed consent document for research participants, ensuring voluntary participation and data protection.
(ZIP)

S1 Data This supplementary file contains qualitative data collected through FGDs and KIIs conducted across Homabay, Bungoma and West Pokot counties in Kenya. The data provide insights from community members, and local experts on the study’s thematic areas. Legend: *Bungoma FGD folder-* Focused group discussion data with Bungoma community members. *Bungoma_PRH folder* -Key Informant interview with patients of religious healers In Bungoma. *Bungoma_PTH folder* -Key Informant interview with patients of traditional healers in Bungoma. *Bungoma_RH folder* -Key Informant interview with religious healers in bungoma. *Homabay FGDs folder* - Focused group discussion data with Homabay community members. *Homabay_PRH folder* -Key Informant interview with patients of religious healers in Homabay. *Homabay_PTH folder* -Key Informant interview with patients of traditional healers in Homabay. *Homabay_TH folder* -Key Informant interview with traditional healers in Homabay. *West Pokot FGDs folder* - Focused group discussion data with West Pokot community members. *West Pokot_PRH folder* -Key Informant interview with patients of religious healers in west Pokot. *West Pokot_TH folder* -Key Informant interview with traditional healers in west Pokot. *West Pokot_TH folder* -Key Informant interview with religious healers in west Pokot.
(RAR)
