# Supplementary material for: The potential role of cultural and religious healing practices in shaping community vulnerability to highly infectious diseases in western Kenya
Source: PLOS Glob Public Health. 2025 Mar 25;5(3):e0003228. doi: 10.1371/journal.pgph.0003228 (PMC11936168; doi:10.1371/journal.pgph.0003228)
Supplement: S1 File — Legend: Guide-for-FGD-Community-Members.docx- Focused Group Discussion (FGD) guide for community members. Guide-for-Religious-Healers.docx; Guide-for-Traditional-Healers.docx- Key Informant Interview (KII) guides for traditional and religious healers. Guide-for-Patient-of-Religious-Healer.docx; Guide-for-Patient-of-Traditional-Healer.docx- Key Informant Interview (KII) guide for patients of traditional and religious healers. Guide-Participatory-Enquiry-Workshop.docx- Participatory workshop guide for stakeholders. Informed-Consent-KRCS.docx- Informed consent document for research participants, ensuring voluntary participation and data protection. (ZIP) [file pgph.0003228.s001.zip › Guide-for-FGD-Community-Members.docx]

FOCUS GROUP DISCUSSION GUIDE FOR COMMUNITY MEMBERS

GENERAL INSTRUCTIONS

Was informed consent obtained?

YES ________ (proceed with interview)

NO ________ (STOP! Thank the participant for their time but do not proceed with the interview)

Moderator’s Name: _______________________ Note-taker’s Name: ________________________

Location of FGD: ________________________ (HOMABAY, BUNGOMA OR WEST POKOT)

1. Interview Date (DD/MM/YYYY) __________________

2. Time Start: _______________________ END time: ___________________

3. Moderator’s initials: _________________________________

**Moderator:**  Read the following statement.

“Thank you for agreeing to participate in this Focus Group Discussion. My name is _____________________________. I am representing the Kenya Red Cross. I will be asking you the questions and my partner ___________________________ will be taking notes during the discussion. We will also be audio taping the group discussions as we speak. The aim of the research is to explore the cultural beliefs, practices, and knowledge systems surrounding health, healing, and disease prevention in Homa Bay, Bungoma and West Pokot counties, with a particular emphasis on the response and interaction of traditional healers, religious institutions, and local tribes/clans in the context of highly infectious diseases like Ebola Virus Disease. By examining these aspects, the study seeks to contribute to a better understanding of local formal healthcare systems to inform strategies for effective highly infectious disease control and prevention. Please feel free to tell us whatever you are comfortable sharing. You should also remember that you do not have to share anything that you are not comfortable sharing and you can discontinue your participation in the study at any time should you wish not to continue.

Focus Group Participants’ Demographic Information

| Code # | Age (years) | Gender (M/F) | Highest Level of Education | Occupation | Role in the Community |
| --- | --- | --- | --- | --- | --- |
|  |  |  |  |  |  |
|  |  |  |  |  |  |
|  |  |  |  |  |  |
|  |  |  |  |  |  |
|  |  |  |  |  |  |
|  |  |  |  |  |  |
|  |  |  |  |  |  |
|  |  |  |  |  |  |
|  |  |  |  |  |  |
|  |  |  |  |  |  |
|  |  |  |  |  |  |
|  |  |  |  |  |  |

**Focus Group Discussion Guiding Questions:**

**Part 1: Infectious Diseases**

1. What is your understanding of highly infectious diseases? Give an example of a highly infectious disease? Please describe the signs and symptoms that a person suffering from the diseases you have mentioned will show.

#**Probes:**

- Severity/seriousness, infectiousness (scale of spread) and mode of transmission for mentioned diseases.
- impact on the community and the health care system

2. In your view, what do you think causes these infectious diseases? (Pay attention to categories of cause i.e. biomedical and supernatural causes of these diseases and cultural beliefs on the causes)

3. What are the common infectious diseases in your community?

- Which among these diseases has this community experienced in the recent past? (when did this happen? describe how it happened? Who were mostly affected - children, women, men?)
- How did the community get to know about the occurrence of this disease? - pay attention to mass media, from public meetings, government announcements, medical campaigns, churches, etc.
- What was done to respond to the outbreak of the infectious diseases you have mentioned? What was done by the households, community, government, public health officers, and police, border controls?
- How quick was the response?
- Was the response effective? Why and how?
- Mention other better ways of response that you think could have helped.

**Part 2: Health Seeking behavior for highly infectious diseases in the community**

**As may be applicable, the facilitator should ask participants to share their life experiences.**

1. If one suffers from highly infectious diseases, like the ones you mentioned earlier, what are the traditional healing practices employed to treat them?

- How is the treatment done - what medicine/treatment is given/taken/where do you get it from?/who prepares it? How is it prepared?
- How is the patient handled - contact, isolation, waste management, the dead etc.?
- Ask how different categories of patients are handled e.g. pregnant women, children under five, women during delivery, the elderly, people with underlying health conditions, people with mental disabilities? Any rituals done to these special group of people?
- When there are many patients, how are they handled?
- How long does the treatment generally take?
- Do people trust the treatment?
- Does it work for them?
- Would you recommend a person to go for that treatment? Why?
- Is there follow-up? How is the follow-up done?
- Why do people choose the traditional healing process for infectious diseases? (cost, flexibility for paying, the way the patient is treated at a formal health facility, effectiveness, testimonials for other people etc.)

1. If one suffers from highly infectious diseases, like the ones you mentioned earlier, what are the religious healing practices employed to treat them?

- How is the treatment done - what medicine/treatment is given/taken/where do you get it from?/who prepares it?
- How is the patient handled - contact, isolation, waste, the dead etc.?
- Ask how different categories of patients are handled e.g. pregnant women, children under five, women during delivery, the elderly, people with underlying health conditions, people with mental disabilities? Any rituals done to these special group of people?
- When there are many patients, how are they handled?
- How long does the treatment generally take?
- Do people trust the treatment?
- Does it work for them?
- Would you recommend a person to go for that treatment? Why?
- Is there a follow-up? How is the follow-up done?
- Why do people choose the traditional healing process for infectious diseases? (cost, flexibility for paying, the way the patient is treated at a formal health facility, effectiveness, testimonials for other people etc.)

1. What other healing practices are employed to treat highly infectious diseases in your communities, other than traditional healing and going to a religious healer?
2. In your community, which is the most preferred treatment for highly infectious diseases? Why?
3. Which of the treatment kinds do people tend to consider first? Why?

**Part 3: Cultural Beliefs and Practices and their Role in the prevention and management of highly infectious diseases, such as Ebola**

1. How are individuals with highly infectious diseases perceived/handled in your community? (**Probes**: Where are they placed? taboos, stigma (any marginalization, discrimination etc.)
2. How do people in your community refer to the Ebola illness? Mention any names/words that people use to refer to Ebola, if any?
3. What do people in your community or places of worship think cause Ebola, and other infectious diseases?
4. Describe cultural rituals and ceremonies or functions in your community you think can enhance the spread of infectious diseases like Ebola? What do you think can be done in such situations to reduce the spread?
5. Describe religious rituals and ceremonies or functions in your community you think can enhance the spread of infectious diseases like Ebola? What do you think can be done in such situations to reduce the spread?
6. In what ways can/do government’s responses to infectious diseases conflict with some important religious or cultural functions/ceremonies/rituals in your community? What can be done to reduce these conflicts?
7. What do you think these categories of people can do in order to prevent the spread of infectious diseases?

- Community members
- Traditional healers
- religious healers
- Health care workers
- community leaders
- Local government administrators
- CBOs and NGOs

**Part 4: Collaboration with and support from formal healthcare system**

1. What external support do you receive during these outbreaks? (NGO, Government, church).
2. What strategies have been put in place to prevent these outbreaks? (probe for community own initiative, government initiatives, CBOs, NGOs)
3. What opportunities are there to enhance collaboration between traditional healers, religious healers and the health care system in prevention and management of highly infectious diseases in the community?
4. What needs to be done differently in terms of treatment and prevention practices for highly infectious diseases?
