# Supplementary material for: The potential role of cultural and religious healing practices in shaping community vulnerability to highly infectious diseases in western Kenya
Source: PLOS Glob Public Health. 2025 Mar 25;5(3):e0003228. doi: 10.1371/journal.pgph.0003228 (PMC11936168; doi:10.1371/journal.pgph.0003228)
Supplement: S1 File — Legend: Guide-for-FGD-Community-Members.docx- Focused Group Discussion (FGD) guide for community members. Guide-for-Religious-Healers.docx; Guide-for-Traditional-Healers.docx- Key Informant Interview (KII) guides for traditional and religious healers. Guide-for-Patient-of-Religious-Healer.docx; Guide-for-Patient-of-Traditional-Healer.docx- Key Informant Interview (KII) guide for patients of traditional and religious healers. Guide-Participatory-Enquiry-Workshop.docx- Participatory workshop guide for stakeholders. Informed-Consent-KRCS.docx- Informed consent document for research participants, ensuring voluntary participation and data protection. (ZIP) [file pgph.0003228.s001.zip › Guide-for-Patient-of-Traditional-Healer.docx]

**KEY INFORMANT INTERVIEW GUIDE FOR PATIENT OF TRADITIONAL HEALERS**

**GENERAL INSTRUCTIONS**

Was informed consent obtained?

YES     ________ (proceed with interview)

NO     ________ (STOP!  Thank the participant for their time but do not proceed with the interview)

Moderator’s Name:  _________________Note-taker’s Name: ________________________

Location of KII: ________________________ (HOMABAY, BUNGOMA OR WEST POKOT)

1.        Interview Date (DD/MM/YYYY)  __________________

2.        Time Start: _______________________ END time: ___________________

3.        Moderator’s initials: _________________________________

Interviewer:  Read the following statement.

“Thank you for agreeing to participate in this interview. My name is _____________________________.  I am representing the Kenya Red Cross. I will be asking you the questions and my partner ___________________________ will be taking notes during the interview. We will also be audio taping the interview as we speak to you. The aim of the research is to explore the cultural beliefs, practices, and knowledge systems surrounding health, healing, and disease prevention in Homa Bay, Bungoma and West Pokot counties, with a particular emphasis on the response and interaction of patients of traditional / religious healers in the context of highly infectious diseases like Ebola Virus Disease. By examining these aspects, the study seeks to contribute to a better understanding of local healthcare systems and inform strategies for effective disease control and prevention.

Please feel free to tell us whatever you are comfortable sharing. You should also remember that you do not have to share anything that you are not comfortable sharing and you can discontinue your participation in the study at any time should you wish not to continue.

**Key Informant’s Demographic Information**

| Code # | Age (years) | Gender (M/F) | Highest Level of Education | Occupation | Role in the Community |
| --- | --- | --- | --- | --- | --- |
|  |  |  |  |  |  |

**Guiding Questions:**

**Part 1: Knowledge about Infectious Diseases**

1. What is your understanding of highly infectious diseases?

- Give an example of a highly infectious disease?
- Please describe the signs and symptoms that a person suffering from the diseases you have mentioned will show.
- Severity/seriousness, infectiousness (Scale of spread) and mode of transmission for mentioned diseases.

2. In your view, what do you think causes these infectious diseases? (Probe for biomedical and supernatural causes of these diseases and cultural beliefs on the causes)

3. What are the common infectious diseases in this community?

- Which among these diseases has this community experienced in the recent past? (when did this happen? describe how it happened? Who were mostly affected - children, women, men?)
- How did the community get to know about the occurrence of this disease? - mass media, from public meetings, government announcements, medical campaigns, churches, etc.
- What was done to respond to the outbreak of the infectious diseases you have mentioned? What was done by the households, community, government, public health officers, and police?
- Was the response effective? Why and how?

**Part 2: Health-seeking behaviors about highly infectious diseases**

1. We have been referred to you because you have been treated by a traditional healer before, is this true? YES/NO

**If NO, thank the participant and stop the interview. Get another relevant participant.**

**If YES, continue with the following probes and questions**

- Did you consult only one or different traditional healers?
- How did you get to know that they could treat these diseases?
- Please tell me which disease you were treated for or describe to me the condition of your sickness for which you consulted each of the traditional healers?.
- What do you think caused those diseases which you consulted the traditional healer for?

1. Please give me reasons why you chose to consult these traditional healers for treatment? **Pay attention to E.g. past experience, advised by a fiend, hospital not near etc?**

- Did you also go to the hospital or clinic to be treated for these diseases? Why?
- If you consulted the hospital, were you given medicine? What did you do with the medicine from the hospital now that you started consulting the traditional healer?

1. Please describe to me the treatment process that you went through while consulting the traditional healer?

- How were you handled? How were you dressed?
- How were the healers dressed? Did they have protective clothing?
- What medicine were you given? How did you use the medicine? E.g. drink, bath, smoke etc? What equipment were you using to take the medicine?
- Did the healer perform any ritual? Which ones?
- Where was the healing taking place? if at home of the healer, were you staying there? Describe to me the sleeping conditions?
- If you were staying at the healers home, were there other people being treated by the same healers? Estimate the number of the patients who were there? What diseases were they suffering from?
- If you were suffering from an infectious disease i.e. could easily spread amongst people, would you still go to the traditional healer? Why?
- What would have done differently if the disease you were treated for by the traditional healer was infectious?
- What do you think the traditional healers should have done if the diseases they were treating you for were infectious?

1. What was the outcome of the treatment you received from the traditional healer?

- Was it effective? Why?
- Were you satisfied? Why do you say so?
- Were you healed?
- Do you think you would consult formal hospital and clinics for similar diseases?

**Part 3: Interaction with the formal healthcare system**

1. If there is an outbreak of an infectious disease that spreads fast amongst people, where do you think people should go for healing? Hospital or traditional healers? Why?
