# Supplementary material for: The potential role of cultural and religious healing practices in shaping community vulnerability to highly infectious diseases in western Kenya
Source: PLOS Glob Public Health. 2025 Mar 25;5(3):e0003228. doi: 10.1371/journal.pgph.0003228 (PMC11936168; doi:10.1371/journal.pgph.0003228)
Supplement: S1 File — Legend: Guide-for-FGD-Community-Members.docx- Focused Group Discussion (FGD) guide for community members. Guide-for-Religious-Healers.docx; Guide-for-Traditional-Healers.docx- Key Informant Interview (KII) guides for traditional and religious healers. Guide-for-Patient-of-Religious-Healer.docx; Guide-for-Patient-of-Traditional-Healer.docx- Key Informant Interview (KII) guide for patients of traditional and religious healers. Guide-Participatory-Enquiry-Workshop.docx- Participatory workshop guide for stakeholders. Informed-Consent-KRCS.docx- Informed consent document for research participants, ensuring voluntary participation and data protection. (ZIP) [file pgph.0003228.s001.zip › Guide-for-Traditional-Healers.docx]

KEY INFORMANT INTERVIEW GUIDE FOR TRADITIONAL HEALERS

GENERAL INSTRUCTIONS

Was informed consent obtained?

YES     ________ (proceed with interview)

NO     ________ (STOP!  Thank the participant for their time but do not proceed with the interview)

Moderator’s Name:  _________________Note-taker Name: ________________________

Location of FGD: _______________(HOMABAY, BUNGOMA OR WEST POKOT)

1.        Interview Date (DD/MM/YYYY)  __________________

2.        Time Start: _______________________ END time: ___________________

3.        Moderator’s initials: _________________________________

Interviewer:  Read the following statement.

“Thank you for agreeing to participate in this interview. My name is _____________________________.  I am representing the Kenya Red Cross. I will be asking you the questions and my partner ___________________________ will be taking notes during the interview. We will also be audio taping the interview as we speak to you. The aim of the research is to explore the cultural beliefs, practices, and knowledge systems surrounding health, healing, and disease prevention in Homa Bay, Bungoma and West Pokot counties, with a particular emphasis on the response and interaction of traditional healers, religious institutions, and local tribes/clans in the context of highly infectious diseases like Ebola Virus Disease. By examining these aspects, the study seeks to contribute to a better understanding of local healthcare systems and inform strategies for effective disease control and prevention.

Please feel free to tell us whatever you are comfortable sharing. You should also remember that you do not have to share anything that you are not comfortable sharing and you can discontinue your participation in the study at any time should you wish not to continue.

Key Informant’s Demographic Information

| Code # | Age (years) | Gender (M/F) | Highest Level of Education | Occupation | Role in the Community |
| --- | --- | --- | --- | --- | --- |
|  |  |  |  |  |  |

**Guiding Questions:**

**Part 1: Knowledge about infectious diseases**

- What is your understanding of highly infectious diseases?
- Give an example of a highly infectious disease?
- Please describe the signs and symptoms that a person suffering from the diseases you have mentioned will show.
- Severity/seriousness, infectiousness (Scale of spread) and mode of transmission for mentioned diseases.

2. In your view, what do you think causes these infectious diseases? (Probe for biomedical and supernatural causes of these diseases and cultural beliefs on the causes)

3. What are the common infectious diseases in this community?

- Which among these diseases has this community experienced in the recent past? (when did this happen? describe how it happened? Who were mostly affected - children, women, men?)
- How did the community get to know about the occurrence of this disease? - mass media, from public meetings, government announcements, medical campaigns, churches, etc.
- What was done to respond to the outbreak of the infectious diseases you have mentioned? What was done by the households, community, government, public health officers, and police?
- Was the response effective? Why and how?

**Part 2: Traditional healing practices for highly infectious diseases in the community**

1. Have you ever handled a patient suffering from the infectious diseases you have mentioned?

- Why do you think these patients came to you?
- What were the signs and symptoms that those patients showed?
- Who brought these patients to you? came by themselves, brought by relatives and friends etc?
- Please describe the conditions that these patients were in when they were brought to you (walking, carried - please describe the mode)?

1. What treatment did you give to the patients? herbal medicine, ritual, massage etc.

- Describe how you administered the treatment/healing practices.

1. How did you handle the patients?

- Please describe how you ensure your safety while handling the patient? (universal precautions - hand washing, wearing protective gears e.g. masks, gloves,)
- How do you handle wastes from the patients?
- How do you handle those who unfortunately die when you are treating them? (any ritual practices, probe on how the rituals are done e.g appeasing the spirits through some rites that may include contact)
- Do you receive more than one patient at a time? Please describe how you handle them?
- How do you handle pregnant woman and delivery of the baby (Probes: Handling of a pregnant mother who has a highly infectious disease; handling and disposal of the placenta; handling of a baby born to mother with highly infectious disease)

1. Have you ever had a patient whose disease you cannot treat? **Give an example**.

- What do you do in such cases?
- Do you refer them to another traditional healer? (specify details), refer to a religious healer (specify details), refer to the hospital (specify details).
- Why do you refer them to those particular places you have mentioned?

1. Have ever handled patients who have or are also going to the hospital for treatment?

- What advice do you give them?
- What treatment do you give them?
- What happens to their medicines they were given at the hospitals? Are there some who stopped using their medicines from the hospital? Why?

1. Where do you see your patients? patients home, healers home, others (describe)

- How do you decide on where to see your patients?

**Part 3: Collaboration, partnership and support from formal healthcare and other stakeholders**

1. Do you receive any support from the government, NGOs or health care workers in line with your healing practices? equipment, education and training etc.

- Who do you receive support from?
- Please describe the kind of support you receive from the stakeholder you have mentioned.
- Mention any other support that you would need from stakeholders towards your healing practices.

1. In what ways do you collaborate with government and other stakeholders in relation to your healing practices?

- Do you report patients with unusual signs and symptoms to government officers? How do you do that? How could this be improved?
- Do you refer patients to formal healthcare? How do you do that?
- How do you report deaths that occur, if any? describe how you do it?

**Part 4: Traditional Beliefs and Practices and their Role in the prevention and management of highly infectious diseases such as Ebola**

1. What do you know about Ebola?

- What do you think causes Ebola?
- What are the signs and symptoms?

1. In your view, do you think you are able to treat Ebola?

- Why do you think you would treat Ebola? Why not?
- How would you treat it?
- If you are not able to treat Ebola, what do you see as appropriate treatments for Ebola?
