# Supplementary material for: The potential role of cultural and religious healing practices in shaping community vulnerability to highly infectious diseases in western Kenya
Source: PLOS Glob Public Health. 2025 Mar 25;5(3):e0003228. doi: 10.1371/journal.pgph.0003228 (PMC11936168; doi:10.1371/journal.pgph.0003228)
Supplement: S1 File — Legend: Guide-for-FGD-Community-Members.docx- Focused Group Discussion (FGD) guide for community members. Guide-for-Religious-Healers.docx; Guide-for-Traditional-Healers.docx- Key Informant Interview (KII) guides for traditional and religious healers. Guide-for-Patient-of-Religious-Healer.docx; Guide-for-Patient-of-Traditional-Healer.docx- Key Informant Interview (KII) guide for patients of traditional and religious healers. Guide-Participatory-Enquiry-Workshop.docx- Participatory workshop guide for stakeholders. Informed-Consent-KRCS.docx- Informed consent document for research participants, ensuring voluntary participation and data protection. (ZIP) [file pgph.0003228.s001.zip › Guide-Participatory-Enquiry-Workshop.docx]

PARTICIPATORY ENQUIRY WORKSHOP GUIDE

GENERAL INSTRUCTIONS

Was informed consent obtained?

YES     ________ (proceed with interview)

NO     ________ (STOP!  Thank the participant for their time but do not proceed with the interview)

Moderator’s Name:  _________________Note-taker’s Name: ________________________

**Location of FGD**: _____________________ (HOMABAY, BUNGOMA OR WEST POKOT)

1.        Workshop Date (DD/MM/YYYY)  __________________

2.        Time Start: _______________________ END time: ___________________

3.        Moderator’s initials: _________________________________

**Moderator**:  Read the following statement.

“Thank you for agreeing to participate in this Participatory Enquiry Workshop. My name is _____________________________.  I am representing the Kenya Red Cross. I will be moderating the workshop and discussions and my colleague___________________________ will be taking notes. The aim of the research is to explore the religious and traditional healing practices, cultural beliefs, practices, and knowledge systems surrounding health, healing, and disease prevention in Homa Bay, Bungoma and West Pokot counties, with a particular emphasis on the response and interaction of traditional healers, religious institutions, and local tribes/clans in the context of highly infectious diseases like Ebola Virus Disease. By examining these aspects, the study seeks to contribute to a better understanding of local healthcare systems to inform strategies for effective disease control and prevention.

Please feel free to tell us whatever you are comfortable sharing. You should also remember that you do not have to share anything that you are not comfortable sharing and you can discontinue your participation in the study at any time should you wish not to continue.

**Participant’s Demographic Information**

| **Code #** | **Age (years)** | **Gender (M/F)** | **Highest Level of Education** | **Occupation** | **Designation/Role** |
| --- | --- | --- | --- | --- | --- |
|  |  |  |  |  |  |
|  |  |  |  |  |  |
|  |  |  |  |  |  |
|  |  |  |  |  |  |
|  |  |  |  |  |  |
|  |  |  |  |  |  |
|  |  |  |  |  |  |
|  |  |  |  |  |  |
|  |  |  |  |  |  |
|  |  |  |  |  |  |
|  |  |  |  |  |  |
|  |  |  |  |  |  |
|  |  |  |  |  |  |
|  |  |  |  |  |  |

1. **Community Health Promoters (CHPs)**

**The Moderator will guide the group to discuss the following questions and take elaborate notes on the responses.**

1. What is your understanding of highly infectious diseases? Give an example of a highly infectious disease? Please describe the signs and symptoms that a person suffering from the diseases you have mentioned will show. Which of these are common in your communities?
2. What is your role in prevention and management of infectious diseases? **(Describe your experiences in each of the roles - challenges and success).**

- Preventing infectious diseases - list the actions you undertake.
- Detection of infectious diseases.
- Detection of highly infectious diseases.
- Response to highly infectious diseases.
- Reporting of highly infectious diseases.
- Referrals - describe the process of doing so.

1. How do religious healing practices (ceremonies, rituals, treatment, handling etc) and traditional healing practices (rituals, treatment, handling etc) **enhance/impede** your roles in preventing and managing infectious diseases? **(Give examples of experiences you have encountered in each case)**
2. Describe, if any, how you work together with traditional and religious healers in the health promotion. **Give examples.**
3. Suggest ways in which the formal healthcare system can better work together with traditional healers and religious healers to enhance prevention and management of highly infectious diseases.

- Preventing infectious diseases - list the actions you undertake.
- Detection of highly infectious diseases.
- Response to highly infectious diseases.
- Reporting of highly infectious diseases.
- Referrals - describe the process of doing so.

1. Suggest strategies that can be used to enhance access to the formal healthcare system by community members infected by highly infectious diseases.
2. Suggest strategies that can be used to prevent and manage outbreaks of highly infectious diseases in your communities.
3. Suggest the training you need in order to enhance your role in prevention and management of highly infectious diseases?
4. **Local government administrators (Chiefs, village elders, border control officials, etc.)**

**The Moderator will guide the group to discuss the following questions and take elaborate notes on the responses.**

1. What is your understanding of highly infectious diseases? Give an example of a highly infectious disease? Please describe the signs and symptoms that a person suffering from the diseases you have mentioned will show. Which of these are common in your communities?
2. What is your role in prevention and management of infectious diseases? **(Describe your experiences in each of the roles - challenges and success).**

- Containment of infectious diseases - list the actions you undertake.
- Detection of highly infectious diseases.
- Response to highly infectious diseases.
- Reporting of highly infectious diseases.
- Referrals - describe the process of doing so.

1. How do religious healing practices (treatment, handling etc) and traditional healing practices (rituals, treatment, handling etc) **enhance/impede** your roles in preventing and managing infectious diseases? **(Give examples of experiences you have encountered in each case).**
2. How do religious practices (ceremonies, functions, rituals, etc) and cultural practices (taboos, values, attitude, rituals, etc) **enhance/impede** your roles in preventing and managing infectious diseases? **(Give examples of experiences you have encountered in each case).**
3. Describe, if any, how you work together with traditional, religious healers, the community and healthcare facilities in the prevention and managing highly infectious diseases in your areas of work. **Give examples.**
4. Suggest ways in which your collaboration with traditional healers, religious healers, the community and the healthcare facilities could be enhanced in the prevention and management of highly infectious diseases.
5. Suggest the training you need in order to enhance your role in prevention and management of highly infectious diseases.
6. **Public Health Professionals & Stakeholders (Public Health Officers, nurses, clinical officers, Medical Officers, local Community Based Organizations and Non-governmental organizations)**

**The Moderator will guide the group to discuss the following questions and take elaborate notes on the responses.**

1. How do religious healing practices (treatment, handling etc) and traditional healing practices (rituals, treatment, handling etc) **enhance/impede** your roles in preventing and managing infectious diseases? **(Give examples of experiences you have encountered in each case).**
2. How do religious practices (ceremonies, functions, rituals, etc) and cultural practices (taboos, values, attitude, rituals, etc) **enhance/impede** your roles in preventing and managing infectious diseases? **(Give examples of experiences you have encountered in each case).**
3. Describe, if any, how you work together with traditional and religious healers in the prevention and management of infectious diseases in your areas of work. **Give examples, including from management of other diseases as well.**
4. What lessons have you learnt from those collaborations practices, projects, programs that can be applied in the prevention and management of highly infectious diseases, like Ebola. **(What can be avoided, what can be adopted?)**.
5. Which approaches have you applied to integrate traditional and religious healing practices in the prevention and management of highly infectious diseases, like Ebola? What worked? What did not work? Why?
6. Suggest the training you would need to enable you to integrate and collaborate better with traditional and religious healers in the prevention and management of highly infectious diseases.
7. List other support that you would need to enable you to integrate and collaborate better with traditional and religious healers in the prevention and management of highly infectious diseases.
