# Supplementary material for: The potential role of cultural and religious healing practices in shaping community vulnerability to highly infectious diseases in western Kenya
Source: PLOS Glob Public Health. 2025 Mar 25;5(3):e0003228. doi: 10.1371/journal.pgph.0003228 (PMC11936168; doi:10.1371/journal.pgph.0003228)
Supplement: S1 File — Legend: Guide-for-FGD-Community-Members.docx- Focused Group Discussion (FGD) guide for community members. Guide-for-Religious-Healers.docx; Guide-for-Traditional-Healers.docx- Key Informant Interview (KII) guides for traditional and religious healers. Guide-for-Patient-of-Religious-Healer.docx; Guide-for-Patient-of-Traditional-Healer.docx- Key Informant Interview (KII) guide for patients of traditional and religious healers. Guide-Participatory-Enquiry-Workshop.docx- Participatory workshop guide for stakeholders. Informed-Consent-KRCS.docx- Informed consent document for research participants, ensuring voluntary participation and data protection. (ZIP) [file pgph.0003228.s001.zip › Informed-Consent-KRCS.docx]

**FGD Informed consent form**

**Title of the Research Study:** Exploring traditional and religious health seeking practices in Western Kenya in the context of infectious diseases like Ebola Virus Disease

**Principal Investigator**

Naomi Ng’ang’a, MSc.

**Co-Principal investigator**

Richard Dimba Kiaka, Ph.D

**Investigators**

Prof. George Ayodo, Ph.D

Damaris A. Ochanda, Ph.D

Sarah Hawi Ngere, MPH

*Study location: Western Kenya (Bungoma, Homa Bay & West Pokot Counties)*

**Introduction**

Greetings to you. My name is _______________________. I would like to invite you to take part in a study that seeks to explore communities’ traditional practices and customs for highly infectious diseases. The study will help us understand communities’ traditional practices and customs for highly infectious diseases in specific contexts. We will assess health seeking behaviors and factors attributed to healthcare seeking behavior. This study will contribute to the planning of interventions to improve health behaviors and designing public health promotion strategies for Ebola Virus Disease and other infectious diseases. Before you decide if you want to take part or not, I invite you to read, or the information below will be read to you, to help you make a decision. Please ask me any questions that you may have as you or I read through this document.

**Invitation**

We invite you to participate in this study because you are a community member who is knowledgeable about customs and traditions on health seeking of your clan/community. Your views will help us understand the community’s traditional practices and customs for highly infectious diseases in your community. This will help contribute to planning of interventions to improve customs and practices during infectious diseases outbreak. You are selected to participate in a group discussion with other community members.

**Study procedure**

The group discussions will include community members who are knowledgeable about customs and traditions on health seeking in their clans/communities. The sessions will take about 1 hour 30 minutes. We will ask you socio demographic information, healthcare seeking customs and practices, reasons behind those practices, among other questions. The discussion will be carried out at a central place which is convenient for all participants and secure. We will ensure privacy and confidentiality while conducting the group discussion.

**Risk associated with the study**

There is minimal risk associated with taking part in this study. You may find some of the questions we may ask to be confidential about you. You have the right to answer or decline to do so. There may be a possibility of your information reaching unintended individuals. However, your privacy is of utmost importance to us, and we will do what we can to protect your information. The only people who will have access to your information will be members of the research team. We will not share your information with anyone else for any reason. Your name or information that can identify you will not be included in any of our reports or publication.

**Benefit of the study**

You will not get any immediate benefit for participating in the study, but if you participate in the study as earlier explained, results from this study will help in understanding context specific healthcare practices and customs for highly infectious diseases like Ebola. This will help contribute to planning of interventions to improve such health practices and customs during highly infectious diseases and improve health outcomes.

**Alternatives to participating in this study**

You do not have to join this study; you can withdraw your participation anytime without the obligation to explain your decision. This will not affect your relationship with the researcher. You can join other research studies if you are eligible.

**Confidentiality**

If you agree to participate in this study, the information that will be collected from you will be used for the purpose of the study only. No direct link will be made between you and the information you provide when reporting or writing about this study. When we publish these results, we will not use your name or identify you personally. Your information will be kept confidential to the extent permitted by law.

**Voluntary participation**

Participation in this study is entirely voluntary. You can choose to withdraw your participation at any time and that will not affect the relationship between you and the researchers, Kenya Red Cross Society or healthcare providers. If you withdraw your participation, we may ask you to willingly share with us reasons for your withdrawal. Such information may help us to improve our approaches and questions. However, you do not have to explain the reason for your withdrawal if you are not comfortable doing so.

**Contact persons**

If you have any concerns about the study or if you do not understand anything, you can ask them now. If you want to ask questions later, please you can call Dr. Richard Kiaka on telephone number **0722391269** or Naomi Ng’ang’a on Telephone number **0705871722**. If you have questions about any ethical issues, you can contact Dr. Philip Ogutu, The Secretariat, Institutional Scientific Ethical Review Committee, Masinde Muliro University of Science and Technology, telephone number 057-2505222/3 or **Mobile: 0710 801 386**.

**Statement of consent: To be completed by participant**

- The consent form has been read out loud to me/or I have read the consent form.
- I have been given the opportunity to ask questions and received satisfactory answers.
- I understand that this project has been reviewed by, and received ethics clearance through the Ethics Review Committee, Masinde Muliro University of Science and Technology.
- I understand that my participation is voluntary, and that I can withdraw my consent at any time without further explanation, and without any adverse consequences.
- I understand that this study involves a discussion about my traditional and religious health seeking practices during infectious diseases outbreak.
- I understand that the outcome of the discussion will not identify my individual person. My information will be held strictly confidential, will not be shared with people outside the research group, will be stored safely, and will be destroyed 7 years after the discussion.
- I understand that only group results, and not my individual results, will be available for scientific and public health purposes.
- I understand that there is no direct benefit for me from being in the study.
- I understand how to raise concerns or make a complaint and who to contact in such a situation.
- I consent with audio recording *(tick what applies):* _____**Yes** ______**No**
- I understand that a copy of the signed consent form will be given to me.
- I agree to take part in the study *(tick what applies):* _____**Yes** ______**No**.

**Consent statement:** I hereby confirm that I have read the information in the consent form, or it has been read to me. I have asked questions pertaining to this and have received satisfactory answers. I have voluntarily given my consent to participate in this study.

Name of Participant: _______________________________________________________________

Signature/Thumbprint: _______________________ Date: _________________________________

**Witness Declaration:** I hereby declare that I have witnessed the reading of the information to the participant who apparently understood everything. The participant was also given the chance to ask questions. I therefore confirm that the participant has given consent freely.

Name of Witness: _____________________________________________________________________

Signature: ________________________ Date: ______________________________________________

**Interviewer statement**: I, the undersigned, have given out the information sheet on the study. I explained the procedures to be followed, risks and benefits involved and answered all questions the participant has about the study. I confirm that the participant has agreed to participate in the study.

Name of Interviewer: __________________________________________________________________

Signature: ________________________ Date: _____________________________________________

**Informed consent form**

**Key Informant Interviews/In-depth interviews/Participatory inquiry workshops**

**Title of the Research Study:** Exploring traditional and religious health seeking practices in Western Kenya in the context of infectious diseases like Ebola Virus Disease

**Principal Investigator**

Naomi Ng’ang’a, MSc.

**Co-Principal investigator**

Richard Dimba Kiaka, Ph.D

**Investigators**

Prof. George Ayodo, Ph.D

Damaris A. Ochanda, Ph.D

Sarah Hawi Ngere, MPH

*Study location: Western Kenya (Bungoma, Homa Bay & West Pokot Counties)*

**Introduction**

Greetings to you. My name is _______________________. I would like to invite you to take part in a study that seeks to explore communities’ traditional practices and customs for highly infectious diseases. The study will help us understand communities’ traditional practices and customs for highly infectious diseases in specific contexts. We will assess health seeking behaviors and factors attributed to healthcare seeking behavior. This study will contribute to the planning of interventions to improve health behaviors and designing public health promotion strategies for Ebola Virus Disease and other infectious diseases. Before you decide if you want to take part or not, I invite you to read, or the information below will be read to you, to help you make a decision. Please ask me any questions that you may have as you or I read through this document.

**Invitation**

We invite you to participate in this study because you are a known:

- Religious healer
- Traditional healer
- Patient or former patient of religious and/or traditional healing practices
- Stakeholder in the management of infectious diseases outbreak in your area of work.

Your views will help us understand the community’s traditional practices and customs for highly infectious diseases in your community. This will help contribute to planning of interventions to improve customs and practices during infectious diseases outbreak. Because of your important role in shaping health seeking behaviors of members of communities in your county, you are selected to participate in:

- Physical in-depth interview.
- Virtual in-depth interview.
- Participatory inquiry workshop.

**Study procedure**

- The physical in-depth interview will last about 1 hour 30 minutes.
- The virtual in-depth interview will last about 1 hour 30 minutes.
- The participatory inquiry workshop will last 3 hours, and will include other stakeholders in the management of infectious diseases outbreak e.g. community health promoters, county health officers and NGOs and CBOs officers and local government administration officers.

We will ask you socio demographic information, healthcare seeking customs and practices, reasons behind those practices, among other questions. The discussion will be carried out at a central place which is convenient for all participants and secure. We will ensure privacy and confidentiality while conducting the group discussion.

**Risk associated with the study**

There is minimal risk associated with taking part in this study. You may find some of the questions we may ask to be confidential about you. You have the right to answer or decline to do so. There may be a possibility of your information reaching unintended individuals. However, your privacy is of utmost importance to us, and we will do what we can to protect your information. The only people who will have access to your information will be members of the research team. We will not share your information with anyone else for any reason. Your name or information that can identify you will not be included in any of our reports or publication.

**Benefit of the study**

You will not get any immediate benefit for participating in the study, but if you participate in the study as earlier explained, results from this study will help in understanding context specific healthcare practices and customs for highly infectious diseases like Ebola. This will help contribute to planning of interventions to improve such health practices and customs during highly infectious diseases and improve health outcomes.

**Alternatives to participating in this study**

You do not have to join this study; you can withdraw your participation anytime without the obligation to explain your decision. This will not affect your relationship with the researcher. You can join other research studies if you are eligible.

**Confidentiality**

If you agree to participate in this study, the information that will be collected from you will be used for the purpose of the study only. No direct link will be made between you and the information you provide when reporting or writing about this study. When we publish these results, we will not use your name or identify you personally. Your information will be kept confidential to the extent permitted by law.

**Voluntary participation**

Participation in this study is entirely voluntary. You can choose to withdraw your participation at any time and that will not affect the relationship between you and the researchers, Kenya Red Cross Society or healthcare providers. If you withdraw your participation, we may ask you to willingly share with us reasons for your withdrawal. Such information may help us to improve our approaches and questions. However, you do not have to explain the reason for your withdrawal if you are not comfortable doing so.

**Contact persons**

If you have any concerns about the study or if you do not understand anything, you can ask them now. If you want to ask questions later, please you can call Dr. Richard Kiaka on telephone number **0722391269** or Naomi Ng’ang’a on Telephone number **0705871722**. If you have questions about any ethical issues, you can contact Dr. Philip Ogutu, The Secretariat, Institutional Scientific Ethical Review Committee, Masinde Muliro University of Science and Technology, telephone number 057-2505222/3 or **Mobile: 0710 801 386**.

**Statement of consent: To be completed by participant**

- The consent form has been read out loud to me/or I have read the consent form.
- I have been given the opportunity to ask questions and received satisfactory answers.
- I understand that this project has been reviewed by, and received ethics clearance through the Ethics Review Committee, Masinde Muliro University of Science and Technology.
- I understand that my participation is voluntary, and that I can withdraw my consent at any time without further explanation, and without any adverse consequences.
- I understand that this study involves a discussion about my traditional and religious health seeking practices during infectious diseases outbreak.
- I understand that the outcome of the discussion will not identify my individual person. My information will be held strictly confidential, will not be shared with people outside the research group, will be stored safely, and will be destroyed 7 years after the discussion.
- I understand that only group results, and not my individual results, will be available for scientific and public health purposes.
- I understand that there is no direct benefit for me from being in the study.
- I understand how to raise concerns or make a complaint and who to contact in such a situation.
- I consent with audio recording *(tick what applies):* _____**Yes** ______**No**
- I understand that a copy of the signed consent form will be given to me.
- I agree to take part in the study *(tick what applies):* _____**Yes** ______**No**.

**Consent statement:** I hereby confirm that I have read the information in the consent form, or it has been read to me. I have asked questions pertaining to this and have received satisfactory answers. I have voluntarily given my consent to participate in this study.

Name of Participant: _______________________________________________________________

Signature/Thumbprint: _______________________ Date: _________________________________

**Witness Declaration:** I hereby declare that I have witnessed the reading of the information to the participant who apparently understood everything. The participant was also given the chance to ask questions. I therefore confirm that the participant has given consent freely.

Name of Witness: _____________________________________________________________________

Signature: ________________________ Date: ______________________________________________

**Interviewer statement**: I, the undersigned, have given out the information sheet on the study. I explained the procedures to be followed, risks and benefits involved and answered all questions the participant has about the study. I confirm that the participant has agreed to participate in the study.

Name of Interviewer: __________________________________________________________________

Signature: ________________________ Date: _____________________________________________
